# miR-150 functions as a tumour suppressor in human colorectal cancer by targeting c-Myb

**DOI:** 10.1111/jcmm.12398

**Published:** 2014-09-18

**Authors:** Junlan Feng, Yongzhi Yang, Peng Zhang, Feng Wang, Yanlei Ma, Huanlong Qin, Yu Wang

**Affiliations:** a Department of Surgery, Shanghai Jiao Tong University Affiliated Sixth People's HospitalShanghai, China; b Department of GI Surgery, Shanghai Tenth People's Hospital Affiliated to Tongji UniversityShanghai, China

**Keywords:** miR-150, c-Myb, colorectal cancer

## Abstract

Our previously published study documented a deregulation of the microRNA miR-150 in colorectal cancer. Here, we investigated further, *in vitro* and *in vivo*, the potential molecular mechanisms underlying the involvement of miR-150 in colorectal cancer, using the appropriate molecular biological methods. We report that miR-150 is a key regulator in the tumourigenesis and progression of colorectal cancer, by acting as a tumour suppressor targeting c-Myb. The current findings suggest that miR-150 may have important roles in the pathogenesis of colorectal cancer.

## Introduction

Colorectal cancer (CRC) is the third most common cause of cancer-related mortality worldwide. The 5-year overall survival rate ranges from 40% to 60% 1,2. Advances in diagnostic and therapeutic approaches have led to excellent expectations for long-term survival of CRC, but there are still a significant proportion of patients who experience relapse and poor outcomes 3–5. A greater understanding of the molecular mechanisms underlying carcinogenesis and progression in CRC would be helpful in improving diagnosis, therapy and prevention.

MicroRNAs (miRNAs) are post-transcriptionally modulate gene expression by negatively regulating the stability or translational efficiency of their target mRNAs 6–8. Accumulating evidence has proved that miRNAs have crucial functions in multiple cellular and biological processes 6,9. Aberrant expression of miRNAs has been reported to be involved in tumourigenesis as oncogenes or tumour suppressors 10,11. Several studies further revealed that miRNA expression signatures are associated with specific tumour subtypes and clinical outcomes 12,13.

Based on miRNA profile screening and clinical confirmation, we previously identified miR-150 as a potential biomarker that could be helpful in diagnosing and determining prognosis in CRC, including response to therapy 14. In the present study, we further describe the biological characteristics and molecular mechanisms of miR-150 in the progression of CRC.

## Materials and methods

### Cell culture

The institutional review board of Shanghai Tenth People's Hospital affiliated with Tongji University, China, approved this project. The human CRC cell line LoVo was obtained from the American Type Culture Collection (Manassas, VA, USA). Cells were cultured in F-12 Ham's medium, supplemented with 10% foetal bovine serum and 1% antibiotic-antimycotic in a humidified 5% CO_2_ atmosphere at 37°C, at a density of 1 × 10^5^ cells/ml.

### Predicting the miRNA targets

Identifying miRNA targets is crucial for investigating miRNA functions. In the present study, two discrete lists of predicted miRNA targets were generated using the computational programs TargetScan (http://www.targetscan.org/) and PicTar (http://pictar.bio.nyu.edu). Then, a third list was created, which contained only those genes predicted by both programs.

### DNA constructs and luciferase target assay

Two c-Myb 3′ untranslated region (UTR) fragments (5′-GTTGCACTTCTTTTTTTGGGAGA-3′ and 5′-GACTGTTTTATAATTTGGGAGT-3′) that included the miR-150 binding site were cloned downstream of the luciferase coding sequence in the pRLTK control vector Promega, Madison, WI, USA to generate pRLTK-c-Myb-WT. In addition, two c-Myb fragments that included a mutated miR-150 binding site (5′-GTTGCACTTCTTTTTTTGGGTCA-3′ and 5′-GACTGTTTTATAATAAGGGTCT-3′) were cloned in parallel as controls (pRLTK-c-Myb-MUT1 and pRLTK-c-Myb-MUT2). A total of 5.0 × 10^4^ cells in 24-well plates were cotransfected with 0.6 μg of the indicated pRLTK Renilla luciferase construct, 60 ng of pGL3 firefly luciferase normalization control, 200 nM of the miR-150 mimics or inhibitor or the corresponding control. The lysates were collected 48 hrs after transfection, and both the Renilla and firefly luciferase activities were measured with a Dual-Luciferase Reporter System (Promega). The experiments were performed in triplicate.

### Immunohistochemistry analysis

After pretreatment of the slides in citrate buffer (pH 6.0), antigen retrieval was performed in a microwave oven for 4 min. For blocking endogenous peroxidase activity, the slides were treated for 10 min. with methanol containing 0.3% hydrogen peroxide. After washing in Tris buffer, slides were incubated with the anti-human c-Myb primary antibody (goat polyclonal, diluted 1:150; Abcam, Cambridge, UK). For immunostaining, a peroxidase-conjugated goat anti-rabbit antibody was used. The remaining procedures were performed in accordance with the manufacturer's instructions.

### Quantitative RT-PCR analysis

Isolation of total RNA from tissues or cultured cells was carried out using Trizol reagent (Invitrogen, Carlsbad, CA, USA) for both miRNA and mRNA analyses. Quantitative RT-PCR of miR-150 was performed with SYBRRPremix Ex Taq miRNA assays (TakaRa, Promega, Madison, WI, USA) in accordance with the manufacturer's instructions, with a 7500 Real-Time PCR system. Small nuclear RNA U6 levels were used as the reference.

Quantitative RT-PCR of c-Myb was performed with a SYBR Green PCR kit (Qiagen, Hilden, Germany) in accordance with the manufacturer's instructions, with glyceraldehyde 3-phosphate dehydrogenase (GAPDH) expression levels as the reference. The relative fold changes of the genes and miRNA expression levels were calculated by the ΔΔCT method, and the values were expressed as 2^−ΔΔCT^. The primers used are shown in Table1.

**Table 1:** Sequences of primers and siRNA used in this study

Name	Sequences
qPCR primers	
miR-150	
Sense	5′-TCTCCCAACCCTTGTACCAGTG-3′
Anti-sense	5′-CAGTGCGTGTCGTGGAGT-3′
c-Myb	
Sense	5′-CTCCGCCTACAGCTCAACTCC-3′
Anti-sense	5′-TCCTTTATTCGCTTTTCCTTCTCA-3′
U6	
Sense	5′-GTGCTCGCTTCGGCAGCACAT-3′
Anti-sense	5′-GTTTAAGCACTTCGCAAGGTA-3′
GAPDH	
Sense	5′-GCATGGCCTTCCGTGTCC-3′
Anti-sense	5′-CCAGCCCCAGCGTCAAAGGTG-3′
RNA oligoribonucleotides	
miR-150 mimics	5′-UCUCCCAACCCUUGUACCAGUG-3′
miR-150 mimics-NC	5′-CAGUACUUUGUGUAGUACAA-3′
miR-150 inhibitor	5′-CACUGGUACAAGGGUUGGGAGA-3′
miR-150 inhibitor-NC	5′-CAGUACUUUGUGUAGUACAA-3′
siRNA	
c-Myb-siRNA	5′-GATCAGAGAGTGATAGAGC-3′
c-Myb-siRNA-NC	5′-GAAGCCAGATCCAGCTTCC-3′

### Western blot

The cells were harvested in RIPA lysis buffer. The tissue samples were also lysed in the RIPsA lysis buffer. The lysates were subjected to 12% SDS-PAGE and transferred to polyvinylidene difluoride membranes (Amersham Biosciences, Little Chalfont, UK).

The membranes were blocked overnight with PBS containing 0.1% Tween-20 in 5% skim milk at 4°C and were subsequently immunoblotted with the primary goat anti-human c-Myb antibody (diluted 1:1000; Abcam). The blots were incubated with the primary antibody for 2 hrs at room temperature. After washing three times in Tris-buffered saline with Tween 20, the blots were incubated with secondary antibody (diluted 1:10,000; Santa Cruz) conjugated to horseradish peroxidase for 2 hrs at room temperature. The blots were visualized using enhanced chemiluminescence reagents (Amersham Pharmacia Biotech, Piscataway, NJ, USA). GAPDH was used as the internal control.

### RNA oligoribonucleotides and cell transfection

Transfection with miR-150 mimic, miR-150 mimic-negative control (NC), miR-150 inhibitor, miR-150 inhibitor-NC (GenePharma, Shanghai, China), c-Myb-siRNA, or c-Myb-siRNA-NC (Invitrogen) was performed with Lipofectamine 2000 reagent (Invitrogen) in accordance with the manufacturers' instructions. The sequences of these RNA oligoribonucleotides are shown in Table1.

### Cell viability assay

Cell growth was determined using a cell counting kit (CCK)-8 assay (Dojindo, Kumamoto, Japan) in accordance with the manufacturer's instructions. LoVo cells were seeded in 96-well plates and transfected with miR-150 mimic, miR-150 mimic-NC, miR-150 inhibitor or miR-150 inhibitor-NC. CCK-8 solution was used to measure cell viability 48 hrs after transfection. The absorbance of each well was measured at 450 nM.

### Flow cytometry analysis

For the cell cycle analysis, 48 hrs after transfection, 1 × 10^6^ cells in the log phase of growth were harvested by trypsinization, washed twice with cold PBS, fixed in ice-cold 70% ethanol, and incubated overnight at −20°C. Propidium iodide (PI, 50 μg/ml; Sigma-Aldrich) and RNAse A (0.1 mg/ml, Sigma-Aldrich) were added to the cells and allowed to stain for 30 min. Then, cells were examined with a fluorescence-activated cell sorting (FACS) flow cytometer (BD Bioscience, San Jose, CA, USA). DNA histograms were analysed with CellQuest software (BD Biosciences).

For the cell apoptosis assay, an Annexin V-fluorescein isothiocyanate (FITC) Apoptosis Detection Kit II (BD Biosciences) was used to measure the membrane redistribution of phosphatidylserine. Cells were treated per the manufacturer's instructions. The pre-labelled cells were detected and apoptosis was quantified using a FACSCalibur flow cytometer with Cell-Quest software (BD Biosciences). AnnexinV-FITC and PI double stain was used to evaluate the percentage of apoptotic cells. AnnexinV^−^ and PI^−^ cells were used as controls. AnnexinV^+^ and PI^−^ cells were considered apoptotic, and AnnexinV^+^ and PI^+^ cells were defined as necrotic. The percentage of cells with apoptotic nuclei (% apoptosis) was calculated. Each test was repeated in triplicate.

### Cell migration and invasion assays

Cell migration and invasion were detected using transwell chambers (8 μm; Corning Costar, Cambridge, MA, USA). The miR-150 mimic, miR-150 mimic control, miR-150 inhibitor and miR-150 inhibitor control LoVo cells (5 × 10^4^) were suspended in 400 μl of serum-free F12K medium and placed in the uncoated (migration assay) or 1:10 diluted Matrigel-coated (invasion assay) upper chamber. The lower chamber was filled with 1 ml complete F12K medium. After an incubation period of 12 hrs at 37°C, the cells on the upper surface of the filter that had migrated to the bottom surface of the membrane were fixed and stained with 0.5% crystal violet solution. The cells on the top surface of the membrane were removed by wiping with a cotton swab. The cells adhering to the bottom surface of the filter that were fixed and stained with 0.5% crystal violet solution were counted in five randomly selected microscopic fields.

### Xenograft experiments

Five micrograms of miR-150 mimic, miR-150 mimic control, miR-150 inhibitor or miR-150 inhibitor control for *in vivo* delivery was dissolved in 30 μl of enzyme-free water and incorporated into 8 μl of transfection reagent (Entranster-*in vivo*, Engreen, China). The mixture was vortexed. Prior to the *in vivo* administration, this preparation was hydrated with 62 μl of PBS at a concentration of 50 μg/ml to achieve the desired dose in 100-μl injections. A volume of 8 μl of transfection agent mixed into 92 μl of PBS (for a total volume of 100 μl) served as the control.

The Ethics Committee for Animal Use of Shanghai Tenth People's Hospital Affiliated to Tongji University approved all the animal experiments. Healthy, male, infertile, 18–20 g, 5-week old nude mice (BALB/C, Shanghai, Chinese Academy of Sciences, China) were subcutaneously injected with LoVo cells (2 × 10^6^/100 μL PBS per mice) in the right flank. After 7 days, the tumour volumes were ∼150 mm^3^.

The mice were randomly divided into six groups (*n* = 5, each), and injected every 2 days for 18 days with PBS (vehicle; 100 μl) or 5 μg/100 μl of miR-150 mimic, miR-150 mimic control, miR-150 inhibitor, miR-150 inhibitor control or transfection agent, respectively.

The tumour volumes were monitored and calculated. The growth curves were plotted using the average tumour volume at set times. The tumour dimensions were recorded for 18 days, after which the mice were killed. The dissected tumours were aliquoted into screw cap tubes in liquid nitrogen and embedded in paraffin after fixation with neutral buffered formalin.

### Terminal deoxynucleotidyl transferase-mediated nick-end labelling (TUNEL) assay

To determine the apoptotic cells in the formalin-fixed, paraffin-embedded tumour sections (5 μm thick), TUNEL staining was performed with the In Situ Cell Death Detection Kit (Roche Diagnostics GmbH, Mannheim, Germany) in accordance with the manufacturer's protocols. The nucleus of the cells was stained with PI. Nuclei with fragmented DNA were visualized under a fluorescence microscope.

### Statistical analyses

The statistical analyses were performed with GraphPad Prism software (Version 5.01; GraphPad, San Diego, CA, USA) and SPSS for Windows version 15.0.0 (SPSS Inc, Chicago, IL, USA). Significance was defined as *P* < 0.05.

## Results

### The role of miR-150 in CRC *in vitro* and *in vivo*

To determine whether miR-150 may function as a tumour suppressor, the effects of miR-150 overexpression or down-regulation on the proliferation of CRC cells were determined *in vitro*. CRC cell line LoVo was transiently transfected with miR-150 mimic, miR-150 mimic-NC, miR-150 inhibitor, miR-150 inhibitor-NC or treated with liposome, or PBS.

**Figure 1:**
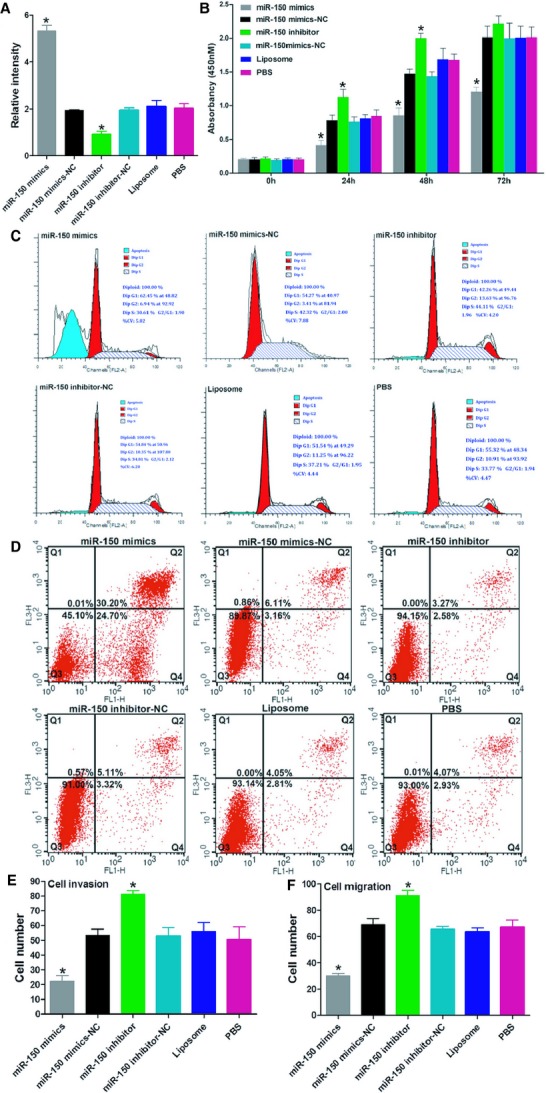
miR-150 regulates proliferation and motility of LoVo cells. (**A**) qRT-PCR assay of miR-150 levels. (**B**) MTT assay for CRC cell proliferative activity. (**C** and **D**) Cell cycle and apoptosis assays showed that miR-150 promoted cell apoptosis and G1 arrest. (**E** and **F**) Transwell assays show effect of miR-150 on cell migration and invasion. Bars represent the mean ± SD of three experiments.

A qRT-PCR assay showed that miR-150 levels were significantly greater in the miR-150 mimic-transfected cells and lower in the miR-150 inhibitor-transfected cells than in the controls (Fig.1A). Results of the CCK-8 assay showed that simulating miR-150 overexpression using a miR-150 mimic led to a significant decrease in LoVo cell proliferation, and that suppressing miR-150 with a miR-150 inhibitor led to a significant increase in LoVo cell proliferation (Fig.1B).

The cell cycle and apoptosis assays revealed that miR-150 promoted cell apoptosis and G1 arrest (Fig.1C and D). In addition, transwell assays found that miR-150 suppressed both migration and invasion in the LoVo cells (Fig.1E and F).

**Figure 2:**
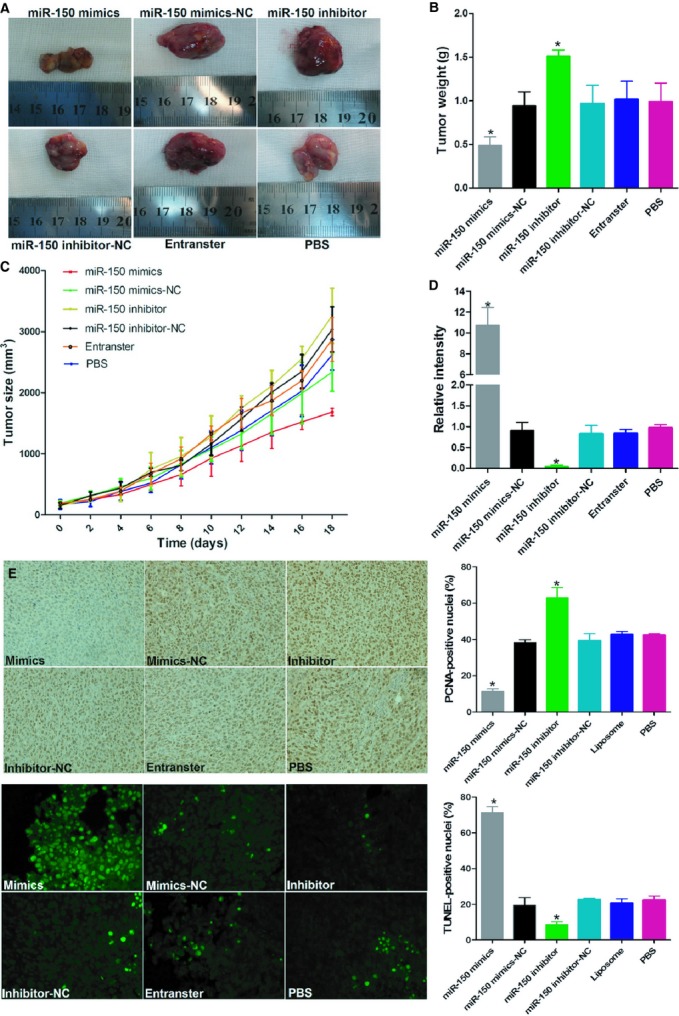
miR-150 regulates tumour growth and apoptosis in CRC xenografts. (**A**) Images of mice bearing LoVo tumours on the 18th day after intra-tumoural injections. (**B**) Tumour weight on the 18th day after intra-tumoural injections. (**C**) Tumour volume growth curve after intra-tumoural injections over the study period. (**D**) qRT–PCR assay of miR-150 levels in different treatment groups. PCNA immunoreactivity (**E**) and TUNEL assay (**F**) for tumour cell proliferation and apoptosis. Bars represent the mean ± SD of three experiments.

To study the anti-oncogene role of miR-150 in CRC, immunodeficient BALB/C mice were subcutaneously injected with LoVo cells. The mice were then treated with an intra-tumoural injection of miR-150 mimic, miR-150 inhibitor or the vehicle. The tumour volumes were measured throughout the treatment course until the animals were killed. Overexpressing miR-150 with a miR-150 mimic significantly decreased tumour growth. Suppressing miR-150 expression with a miR-150 inhibitor significantly promoted tumour growth *in vivo*, with no apparent toxicity at the doses used (Fig.2A–D). A proliferating cell nuclear antigen (PCNA) immunoreactivity analysis and a TUNEL assay found that miR-150 suppressed tumour cell proliferation and promoted cell apoptosis *in vivo* (Fig.2E and F). Taken together, these data indicate that miR-150 has an important role in CRC progression.

### miR-150 can down-regulate c-Myb by targeting its 3′-UTR

To explore the mechanism by which miR-150 suppresses tumour progression, we used two algorithms that predict mRNA targets: miRNA-PicTar, and TargetScan. Based on the frequencies of miR-150 sites in their 3′-UTRs, >100 mRNAs were predicted to be regulated by miR-150. Guided by this gene ontology analysis, c-Myb was among the top target genes predicted for miR-150.

**Figure 3:**
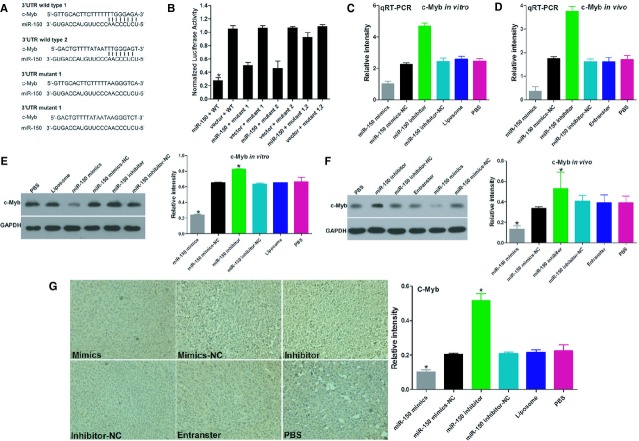
miR-150 directly targets the c-Myb gene *in vitro* and *in vivo*. (**A**) The putative miR-150-binding site in the c-Myb 3′-UTR. (**B**) The miR-150-binding site in the c-Myb 3′-UTR was confirmed in the LoVo cells using a cotransfection luciferase assay for the indicated reporters and the miR-150 mimic or for the indicated reporters and the miR-150 inhibitor. (**C** and **E**) qRT-PCR and Western blot analysis of *in vitro* c-Myb levels. (**D** and **F**) c-Myb mRNA and protein levels were determined by qRT-PCR and Western blot analysis after injecting the miR-150 mimic, mimic control, miR-150 inhibitor or inhibitor control into established LoVo CRC xenografts. (**G**) Immunohistochemistry assay for c-Myb immunoreactivity. Bars represent the mean ± SD of three experiments.

To determine whether c-Myb is regulated by miR-150 *via* direct binding of its 3′-UTR, we constructed c-Myb mRNA 3′-UTR fragments (either wild-type or mutant) and inserted them immediately downstream of the luciferase reporter gene (Fig.3A). In the luciferase assays, the miR-150 mimic was cotransfected into the LoVo cells using different 3′-UTR luciferase constructs. miR-150 decreased the relative luciferase activity in the wild-type 3′-UTR of c-Myb. Further analysis showed that such regulation was sequence-specific, as relative luciferase activities did not decrease as sharply in the UTRs with mutant binding sites as they did in their wild-type counterparts (Fig.3B).

We next evaluated the mRNA and protein-level effects of miR-150 overexpression and inhibition on c-Myb expression in LoVo cells using qRT-PCR and Western blot (Fig.3C and E). Transfecting the LoVo cells with a miR-150 mimic significantly decreased the c-Myb mRNA and protein levels, whereas transfecting the LoVo cells with a miR-150 inhibitor significantly increased the c-Myb mRNA and protein levels.

In an *in vivo* experiment with a tumour xenograft model that used LoVo cells, qRT-PCR and Western blot analysis found that c-Myb expression at the mRNA and protein levels decreased in the miR-150 mimic-treatment group and increased in the miR-150 inhibitor-treatment group, relative to the controls (Fig.3D and F). c-Myb immunoreactivity was readily detected in the cytoplasm and occasionally in the nucleus. The c-Myb staining intensity decreased in the miR-150 mimic-treatment group and increased in the miR-150 inhibitor-treatment group relative to the controls (Fig.3G). Taken together, these results suggest that miR-150 down-regulates c-Myb expression in large part by directly targeting its 3′-UTR.

### c-Myb can block the effects of miR-150 on CRC cells

**Figure 4:**
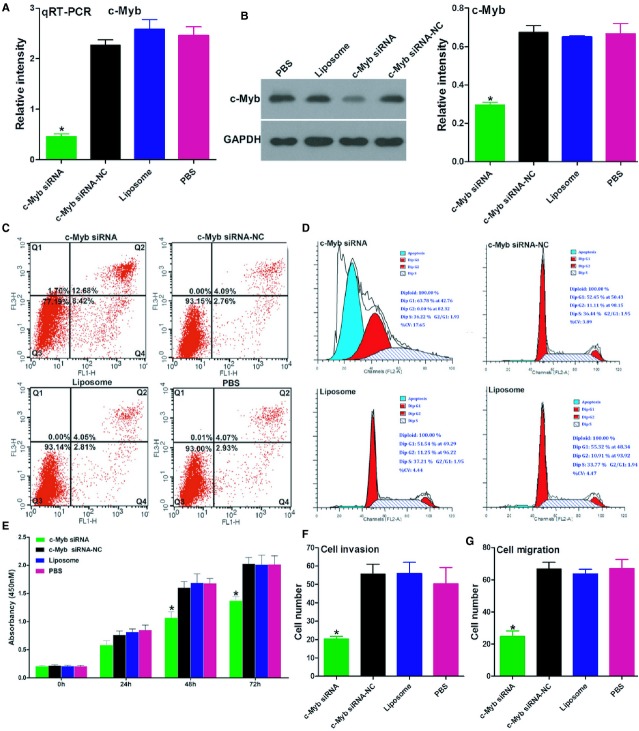
c-Myb-siRNA can partly mimic the effects of miR-150 in LoVo cells. (**A** and **B**) Efficacy of RNA interference for c-Myb, verified by qRT-PCR and Western blot analysis in LoVo cells. (**C** and **D**) Cell cycle and apoptosis assays showed that down-regulating c-Myb significantly promoted apoptosis and G1 arrest in the LoVo cells 48 hrs after transfection. (**E**) Down-regulation of c-Myb significantly inhibited the *in vitro* growth of LoVo cells in an MTT assay. (**F** and **G**) The transwell assay showed that c-Myb knockdown markedly inhibited the invasive and migratory potential of LoVo cells. Bars represent the mean ± SD of three experiments.

To assess the functional contributions of these miR-150 targets to the aggressive phenotypes of the cancer cells, we first examined the role of c-Myb on cell-cycle progression and tumourigenesis in the LoVo cells by knocking down c-Myb with siRNA. The qRT-PCR and Western blot assays showed that c-Myb mRNA and protein levels were significantly inhibited in c-Myb-transfected cells relative to the controls (Fig.4A and B). Notably, the apoptosis, cell-cycle, CCK-8 and transwell assays showed that, similar to the effects of miR-150 overexpression, c-Myb-siRNA was associated with significantly increased levels of cell apoptosis, decelerated cell cycle progression and proliferation, and inhibited invasive and migratory abilities in the LoVo cells *in vitro* (Fig.4C–G).

**Figure 5:**
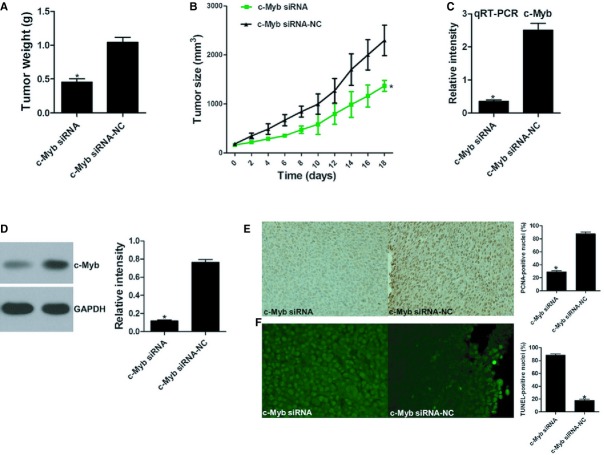
c-Myb regulates tumour growth and apoptosis in CRC xenografts. (**A**) Images of mice bearing LoVo tumours on the 18th day after intra-tumoural injections. (**B**) Tumour weight on the 18th day after intra-tumoural injections. (**C**) Tumour volume growth curve after intra-tumoural injections over the study period. (**D**) and (**E**) qRT-PCR and Western blot assay for c-Myb levels. PCNA immunoreactivity (**F**) and TUNEL assay (**G**) for tumour cell proliferation and apoptosis. Bars represent the mean ± SD of three experiments.

To further study the oncogene role of c-myb in CRC, immunodeficient BALB/C mice were subcutaneously injected with LoVo cells. The mice were then treated with an intra-tumoural injection of a c-Myb-siRNA or a control. The tumour volumes were measured throughout the treatment course until the animals were killed. Suppressing c-Myb expression with siRNA significantly decreased tumour growth *in vivo*, with no apparent toxicity at the doses used (Fig.5A and B). qRT-PCR and Western blot assays showed that c-Myb mRNA and protein levels were significantly inhibited in the c-Myb-siRNA group compared with the controls (Fig.5C and D). A PCNA immunoreactivity analysis and a TUNEL assay found that c-Myb-siRNA suppressed tumour cell proliferation and promoted cell apoptosis *in vivo* (Fig.5E and F). The above evidence indicates that c-Myb is indeed a functional target of miR-150.

## Discussion

Although in our previously study 14 miR-150 was identified as a potential biomarker in CRC for diagnosing and predicting survival, including response to therapy, the molecular mechanisms by which miR-150 modulate the process of carcinogenesis remain obscure. In this study, the data indicate that reintroduction of miR-150 dramatically repressed proliferation of human CRC cells *in vitro*, and induced cell apoptosis and inhibited cell migration and invasion. We also observed the effects of miR-150 in a tumour xenograft model. It is noteworthy that, consistent with the *in vitro* assay results, increasing miR-150 *in vivo* was associated with less tumour growth, inhibition of cell proliferation and greater rates of apoptosis. Consequently, this is clear indication that miR-150 is a promising novel CRC therapeutic target that offers an attractive approach for further investigation. Our findings also support a causal role for altered miR-150 expression during tumourigenesis.

Each miRNA can potentially down-regulate many target genes through binding their 3′-UTR 6–8. As the specific binding of miRNA and target genes depend on cell types and microenvironment, miRNA in different tissues or cancers may have different roles 15. One important task is to identify the downstream target genes regulated by the dysregulated miRNA. By using two algorithms (miRNA-PicTar, and TargetScan), a candidate downstream target gene of miR-150, c-Myb, was identified. c-Myb is frequently overexpressed in many malignancies, and has a crucial role in promoting cell proliferation, survival and metastasis 16–19. However, except for rarely reported amplification 20, gene truncation 21 and mutation 22, the mechanisms of c-Myb activation in cancer remain unclear.

In the present study, quantitative PCR and Western blot analysis showed that miR-150 could decrease c-Myb at both the mRNA and protein levels. In addition, inhibiting miR-150 expression was associated with an increase in c-Myb. The luciferase assay showed that miR-150 could interact with the 3′-UTR of c-Myb. We also demonstrated that, *in vitro*, suppressing c-Myb inhibited cell proliferation, induced cell apoptosis and inhibited cell migration and invasion in human CRC cells. These data strongly suggest that the tumour-suppressive function of miR-150 is by way of down-regulation of c-Myb in the progression of CRC.

In summary, we demonstrated in this study that the tumour-suppressing character of miR-150 functions by repressing its downstream target gene c-Myb, and down-regulation of miR-150 is one of the molecular mechanisms that leads to the development and progression of CRC.
