# Corrigendum

**DOI:** 10.1111/jcmm.17503

**Published:** 2022-09-11

This article corrects the following:

Junlan Feng, Yongzhi Yang, Peng Zhang, Feng Wang, Yanlei Ma, Huanlong Qin, Yu Wang. miR‐150 functions as a tumour suppressor in human colorectal cancer by targeting c‐Myb. J Cell Mol Med. 2014 Oct; 18(10):2125–34. doi: 10.1111/jcmm.12398.

In Junlan Feng et al,^1^ there is an image assembly error in the Mimics group in Figure 2E. The correct figure is shown below. The authors confirm that all results and conclusions of this article remain unchanged.

**FIGURE 2:**
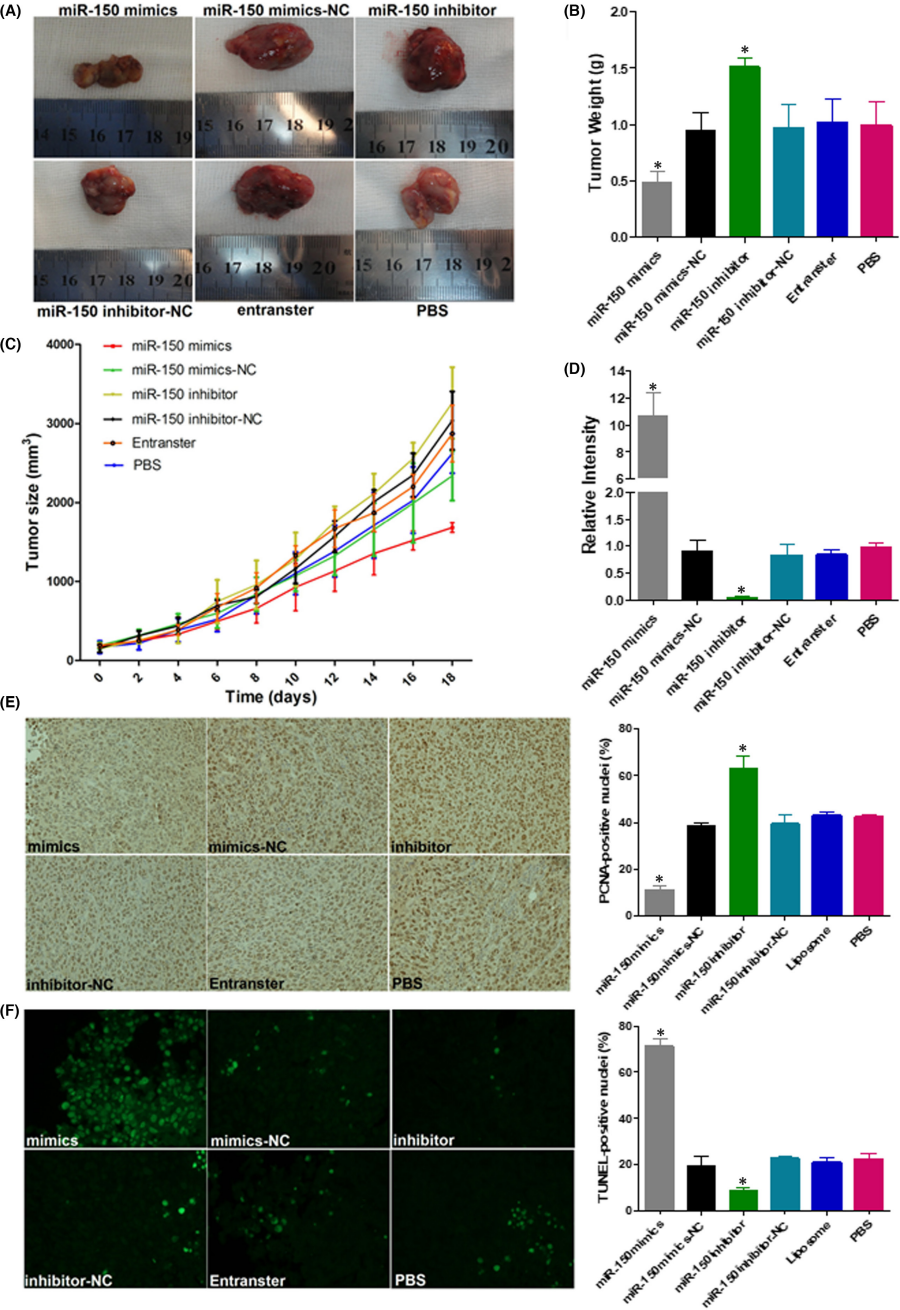
miR‐150 regulates tumour growth and apoptosis in CRC xenografts. (A) Images of mice bearing LoVo tumours on the 18th day after intra‐tumoral injections. (B) Tumour weight on the 18th day after intra‐tumoral injections. (C) Tumour volume growth curve after intra‐tumoral injections over the study period. (D) qRT‐PCR assay of miR‐150 levels in different treatment groups. PCNA immunoreactivity (E) and TUNEL assay (F) for tumour cell proliferation and apoptosis. Bars represent the mean ± SD of three experiments.
